# Troponin I levels in systemic sclerosis patients with myocardial involvement

**DOI:** 10.1177/23971983241255550

**Published:** 2024-06-03

**Authors:** Eva M Hoekstra, Sophie IE Liem, Saad Ahmed, Nivine Levarht, Cynthia M Fehres, Adrian Giuca, Nina Ajmone Marsan, Tom WJ Huizinga, Jeska K de Vries-Bouwstra

**Affiliations:** 1 Department of Rheumatology, Leiden University Medical Center, Leiden, The Netherlands; 2 Department of Cardiology, Leiden University Medical Center, Leiden, The Netherlands

**Keywords:** Systemic sclerosis, myocardial involvement, troponin

## Abstract

**Objectives:**

Troponin I has been suggested as a more specific diagnostic biomarker for myocardial involvement in systemic sclerosis than the frequently used troponin T. The aim of this study is to evaluate the additive value of troponin I to detect myocardial involvement in systemic sclerosis. To this end, we evaluated the association between troponin I levels and myocardial involvement in systemic sclerosis patients.

**Methods:**

A cross-sectional observational study was performed, including 20 healthy controls and four groups of each 20 systemic sclerosis patients from the Leiden Combined Care in Systemic Sclerosis cohort: (1) patients with myocardial involvement, (2) patients with myositis, (3) patients with elevated troponin T and creatine kinase levels but without organ involvement, and (4) patients without any signs of organ involvement. Troponin I levels were measured using enzyme-linked immunosorbent assay. Troponin I levels were compared between the different groups using the Mann–Whitney *U* and Kruskal–Wallis tests.

**Results:**

The mean age of the 80 included patients was 56 years; 61% of the study population was female. Troponin I levels were not significantly different between patients with and without myocardial involvement (2.7 (0.5–15.3) vs 1.2 (0.1–6.6) ng/L; p = 0.117). Systemic sclerosis patients were more often positive for troponin I than healthy controls (70.0% vs 30.0%; p = 0.001).

**Conclusion:**

Elevated troponin I was not of additional value to diagnose myocardial involvement in systemic sclerosis patients.

## Introduction

Systemic sclerosis (SSc) is a chronic connective tissue disease, that is characterized by vascular dysfunction and fibrosis of the skin and internal organs including cardiac involvement.^1^ Disease severity of SSc is reflected by a standardized mortality ratio of 2.78, with cardiac involvement being one of the main factors associated with worse outcome and mortality.^2–4^

Cardiac involvement is clinically apparent in approximately 10%–30% of SSc patients.^5^ Subclinical cardiac involvement is even estimated at >70%, depending on utilized diagnostic screening tools.^6^ In contrast to other SSc-related complications, a definition of myocardial involvement that has been validated with prospective studies is not available. An expert-based consensus on the definition of primary heart involvement has recently been published and highlights the challenge in defining and identifying primary heart involvement in SSc patients.^7^ Since SSc-related heart involvement importantly impacts prognosis, early recognition of cardiac involvement is important.^8^

Currently, endomyocardial biopsy (histological confirmation of myocardial fibrosis) is the most specific examination available.^9^ Nonetheless, its applicability in clinical practice is limited because of the invasive nature of the test and the observation that histological-proven heart involvement does not necessarily correlate with clinically meaningful myocardial involvement.^9,10^ Another frequently used biomarker for myocardial damage is troponin T (TnT).^11,12^ However, the usefulness of this biomarker in the diagnosis of cardiac involvement in SSc is still unclear, as elevated TnT levels can also be found in patients with peripheral muscular damage without the presence of cardiac disease. Recent studies showed that troponin I (TnI) might be more specific for involvement of the myocardium. For example, in patients with muscle injury, renal injury or idiopathic inflammatory myopathies elevated TnT, but normal TnI levels were found.^13^ As a consequence, TnI could be of additive value in identifying patients with possible SSc-associated myocardial involvement and thus in need of more extensive diagnostic testing.^14^

A recent study demonstrated that high-sensitivity troponin I (hs-TnI) was associated with echocardiographic abnormalities in SSc patients.^15^ On the contrary, Hromádka et al.^16^ found no association between hs-TnI and cardiac magnetic resonance parameters in 33 asymptomatic SSc patients. A systematic review on the usefulness of both troponins and B-type natriuretic peptides concluded that there might be a role for troponins in identifying SSc-related myocardial involvement, but no definite conclusions could be made due to the lack of data resulting from the heterogeneity in the included studies.^17^

The primary aim of this study is therefore to evaluate the association between TnI levels and myocardial involvement in SSc patients and to determine whether including TnI measurement in clinical practice is of additional help next to TnT measurements to identify SSc patients with myocardial involvement.

## Methods

### Study design

This was a cross-sectional observational study including patients from the Leiden Combined Care in Systemic Sclerosis (CCISS) cohort. Patients in the CCISS cohort undergo annual screening, including physical examination, laboratory testing, and cardiopulmonary screening. In addition, blood samples are collected annually and stored in the Biobank Rheumatic Diseases of Leiden University Medical Center (LUMC). All patients who are registered in the CCISS cohort gave written informed consent to participate in studies concerning the Biobank Rheumatic diseases. The Biobank Rheumatic diseases (CME no. B16.037, REU 043/SH/sh), the CCISS cohort (P09.003), and this study (B21.078) have previously been approved by the LUMC Committee of Medical Ethics.

### Patients

Inclusion criteria for this study were age ⩾18 years, and a diagnosis of SSc according to American College of Rheumatology/European League Against Rheumatism (ACR/EULAR) 2013 classification criteria.^18^ Moreover, we sought to include four different subgroups of SSc patients: (1) patients with primary myocardial involvement, (2) patients with myositis, (3) patients with elevated TnT levels (>14 ng/L) without other abnormalities indicating organ involvement including and specifically no signs of myocardial involvement (normal electrocardiogram (ECG), Holter monitor, echocardiography, and no cardiac symptoms), and (4) patients with normal TnT levels without any organ involvement including and specifically no signs of myocardial involvement.

Primary myocardial involvement was assessed independently by an experienced rheumatologist and an experienced cardiologist using a score form (see Supplementary File S1). In case of discrepancies in agreement, the case was discussed to achieve consensus. In case of persisting discrepancies in agreement, the patient was excluded from the study. Preferably, patients who underwent cardiac magnetic resonance imaging (MRI) were selected. Myositis was defined as increased creatine kinase (CK) and muscle weakness, preferably confirmed with biopsy. Organ involvement was defined as the presence of any of the following: interstitial lung disease (as determined by high-resolution computed tomography (HRCT) or abnormal X-ray with a forced vital capacity lower than 70% of predicted), critical digital ischemia (such as digital necrosis, gangrene, or amputation), pulmonary arterial hypertension (confirmed with right heart catheterization or diagnosed and treated by a cardiologist), renal crisis (i.e. a new onset of hypertension >150/85 mm Hg obtained at least twice over a consecutive 24-h period or rapid decline in eGFR), myositis (defined as above), or death at any time during follow-up.

In addition, 20 samples from healthy controls (from the LUMC voluntary donors service (LuVDS)), matched for age and sex, were included. A sample size calculation was performed with a significance level of 5% (p < 0.05) and a detection power for myocardial involvement of 80%. As sensitivity and specificity of TnI to detect cardiac involvement is not available, we used the sensitivity and specificity as known for the diagnosis of myocardial infarction.^19^ Based on these data and the prevalence of primary myocardial involvement as demonstrated by Desai et al.,^5^ a sample size of at least 13 samples with cardiac involvement was calculated. We strived to include 20 patients with clear cardiac involvement.

### Materials and TnI analysis

TnI levels were measured in patients’ sera using a RayBio^®^ Human Cardiac Troponin I enzyme-linked immunosorbent assay (ELISA). The assay was performed following the manufacturer’s protocol. Sera were selected from study visits in which there was specific organ involvement according to the inclusion criteria. A positive sample was defined as a TnI value >0.38 ng/L (i.e. the minimal detectable dose of human TnI according to the manufacturer’s protocol).

The standard curve calculated with the RayBio^®^ Human Cardiac Troponin I kit reached a maximum OD of 2.3, corresponding to a TnI level of 341.6 ng/L. Therefore, we manually set back all samples with OD >2.3 to OD 2.3 (corresponding to a TnI level of 341.6 ng/L).

### Disease outcomes

The following disease characteristics were collected for all patients at the time of the serum collection: age, sex, comorbidity (including cardiovascular disease and diabetes mellitus), smoking, disease duration since first non-Raynaud symptom, auto-antibody status (anti-centromere (ACA) and anti-topoisomerase I (ATA) positivity), disease subset (non-cutaneous, limited cutaneous, or diffuse cutaneous), forced vital capacity percentage of predicted (FVC), interstitial lung disease (ILD) defined as ILD on CT scan combined with FVC <80%, creatinine, CK, TnT, and use of immunosuppressive medication.

### Statistical analysis

To summarize clinical and serological features, descriptive statistics were used, and potential differences between groups were tested as appropriate. Median TnI levels were compared between patients with or without primary myocardial involvement using a Mann–Whitney *U*-test. A chi-square test was used to test differences in proportion of positive test between SSc patients and healthy controls. TnI levels in the four subgroups of patients were compared using a Kruskal–Wallis test. Correlation between TnI and TnT levels was measured using Spearman’s rank correlation. All performed tests were two-sided, and p-values < 0.05 were considered as statistically significant. Statistical analysis was performed using IBM SPSS version 25.

## Results

### Patient characteristics

Eighty patients were included. Of the 20 SSc patients with primary myocardial involvement, 15 patients had an available cardiac MRI. Myositis was confirmed with a biopsy in 11 of 20 SSc patients with myositis.

The clinical characteristics of the included patients are shown in Table 1, and the clinical characteristics of the four different subgroups are shown in Supplementary File S2. The mean age was 58.6 years, 61.3% was female, and median disease duration at time of sample collection was 4.6 years. SSc patients with myocardial involvement more often had cardiovascular disease (35.0% vs 17.4%) and diffuse cutaneous SSc (80.0% vs 33.3%), and were less often ACA positive (0 vs 35.0%).

**Table 1.** Patient characteristics of included patients with systemic sclerosis at time of sample collection.

	SSc patients (n = 80)	SSc patients with myocardial involvement (n = 20)	SSc patients without myocardial involvement (n = 60)
Age (years)	58.6 ± 13.1	54.6 ± 13.8	60.0 ± 12.6
Female sex, *n (%)*	49 (61.3)	13 (65.0)	36 (60.0)
Smoking (at time of study visit), *n (%)*	10 (12.5)	1 (5.0)	9 (15.0)
Comorbidity:			
Cardiovascular disease, *n (%)*	14 (17.4)	7 (35.0)	7 (11.7)
Diabetes mellitus, *n (%)*	5 (6.3)	1 (5.3)	4 (6.7)
Disease duration^^^ (years)	4.7 (1.6-11.9)	6.6 (1.3-14.0)	4.3 (1.6-9.3)
Diffuse SSc, *n (%)*	36 (45.0)	16 (80.0)	20 (33.3)
ANA, *n (%)*	78 (97.5)	18 (90.0)	60 (100)
Anti-topoisomerase I, *n (%)*	20 (25.0)	8 (40.0)	12 (20.0)
Anti-centromere, *n (%)*	21 (26.3)	0 (0)	21 (35.0)
Troponin T (ng/L)^~^	23.0 (8.0-56.0)	26.0 (10.5-108.3)	22.0 (8.0-49.5)
Creatinine (μmol/L)	76.5 (63.5-97.3)	80.0 (58.0-86.0)	75.0 (66.0-100.0)
CK (U/L)	109.0 (71.8-197.8)	103.0 (72.0-283.0)	115.0 (71.0-187.0)
ILD^#^, *n (%)*	12 (15.0)	4 (20.0)	8 (13.3)
PAH, *n (%)*	2 (2.5)	1 (5.0)	1 (1.7)
Use of any immunosuppressants ever^*^, *n (%)*	55 (68.8)	13 (65.0)	42 (70.0)

ANA: anti-nuclear antibody; ATA: anti-topoisomerase antibody; ACA: anti-centromere antibody; CK: creatinine kinase; ILD: interstitial lung disease; PAH: pulmonary arterial hypertension.

Parametric data are reported as mean ± SD, non-parametric data as median (IQR).

^ Since first non-Raynaud symptom.

* Includes methotrexate, azathioprine, cyclophosphamide, mycophenolate mofetil, corticosteroids, and biologicals.

~ Note that 40 out of 80 patients were selected partially based on elevated or normal troponin T levels.

# Defined as ILD on CT combined with FVC <80%.

### TnI levels

**Figure 1.**
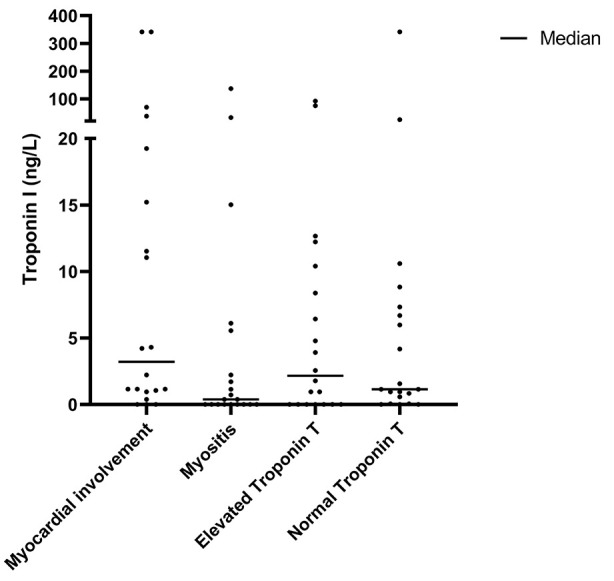
Troponin I levels in subgroups of SSc patients.

Median TnI and TnT levels in SSc patients are presented in Table 2; individual TnI values of the included SSc patients are displayed in Figure 1. TnI levels measured in all included SSc patients ranged from 0 to 341.6 ng/L. The median TnI level in SSc patients with myocardial involvement was 3.2 (1.0–18.2) ng/L and in SSc patients without myocardial involvement 1.0 (0.0–6.6) ng/L (p = 0.068). SSc patients with myositis, SSc patients with elevated TnT levels without organ involvement, and SSc patients with normal TnT levels without organ involvement had median TnI levels of 0.4 (0.0–4.7), 2.2 (0.0–9.9), and 1.2 (0.2–7.2) ng/L, respectively. No significant differences were found in median TnI levels between the 4 different subgroups (p = 0.131).

**Table 2.** Troponin levels in subgroups of SSc patients.

	Primary myocardial involvement (n = 20)	Myositis (n = 20)	No organ involvement (elevated troponin T) (n = 20)	No organ involvement (normal troponin T) (n = 20)	p-value
Troponin I (ng/L)	3.2 (1.0-18.2)	0.4 (0.0-4.7)	2.2 (0.0-9.9)	1.2 (0.2-7.2)	0.131^*^
Troponin T (ng/L)	26.0 (10.5-108.3)	122.0 (17.5-457.5)	31.5 (23.5-46.0)	7.0 (4.0-10.0)	-

* Kruskal-Wallis test.

### Correlation between TnT and TnI

**Figure 2.**
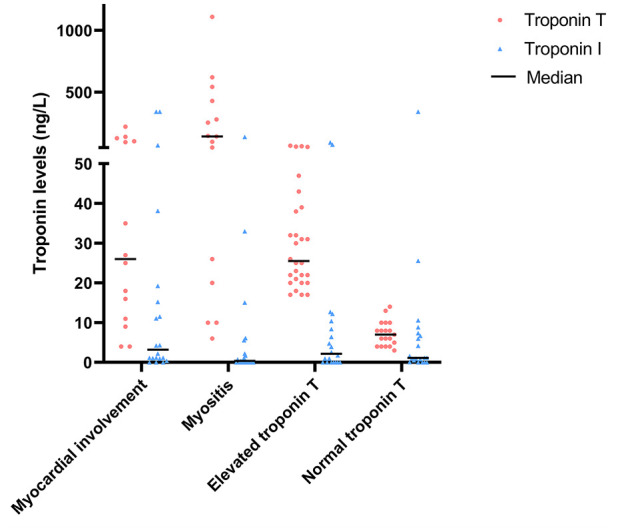
Troponin I and T levels in subgroups of SSc patients.

Median TnT levels in SSc patients with and without myocardial involvement were comparable (26.0 (10.5–108.3) vs 22.0 (8.0–49.5) ng/L, respectively, p = 0.527). In both SSc patients with and without myocardial involvement, TnT and TnI were not significantly correlated with each other (Spearman’s rank correlation coefficient: −0.020 (p = 0.946) and −0.027 (p = 0.849), respectively) (Table 3). In Figure 2, the levels of TnT and TnI in the different subgroups of SSc patients are shown. A wide range of TnT levels were observed in the myositis group (interquartile range (IQR) = 17.5–457.5 ng/L), while the range of TnI levels in this subgroup is smaller (IQR = 0.0–4.7 ng/L).

**Table 3.** Correlation between troponins in patients with systemic sclerosis.

	Primary myocardial involvement (n = 20)	No primary myocardial involvement (n = 60)
Troponin T (ng/L)	26.0 (10.5–108.3)	22.0 (8.0–49.5)
Troponin I (ng/L)	3.2 (1.0–18.2)	1.0 (0.0–6.6)
Spearman’s rank correlation:	−0.020	−0.027
Correlation coefficient	p = 0.946	p = 0.849

### TnI in healthy controls

**Figure 3.**
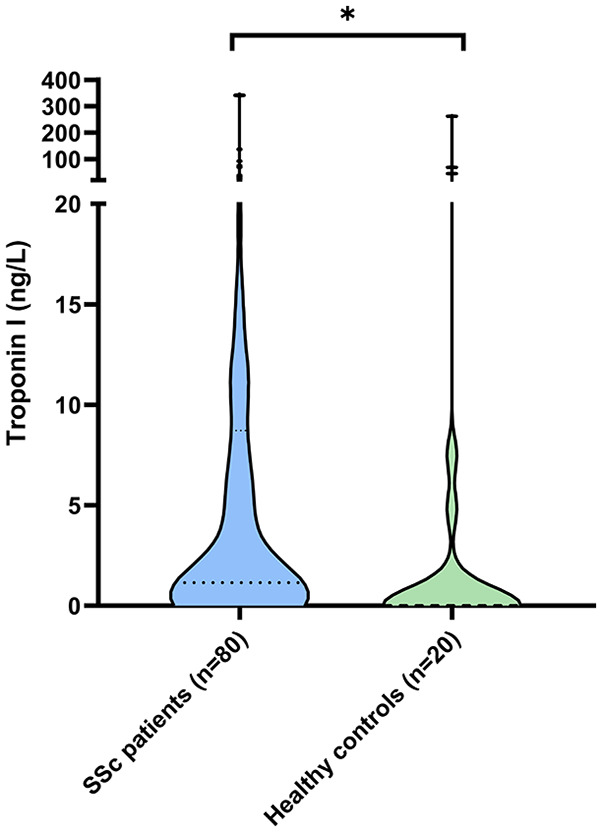
Troponin I levels in SSc patients and healthy controls.

Twenty healthy controls (mean age = 55 ± 13 years, 60% female) were analyzed. Healthy controls were significantly less often positive for TnI compared to the total group of SSc patients (30.0% vs 70.0%, p = 0.001), as well as in the subgroups of patients with and without myocardial involvement (30.0% vs 85.0%; p < 0.001 and 30.0% vs 65.0%; p = 0.006, respectively). The distribution of TnI levels across healthy controls and SSc patients is displayed in Figure 3.

## Discussion

In this cross-sectional observational study, we observed similar TnI levels in SSc patients with and without myocardial involvement. Compared to healthy controls, SSc patients were more often positive for TnI. However, TnI—on its own—was not discriminative to identify SSc patients with myocardial involvement. TnT was also not specific for myocardial involvement and showed highly elevated levels especially in SSc patients suffering from myositis. TnI did not correlate with TnT.

The results of this study are in contrast to earlier research describing an association between TnI and echocardiographic abnormalities in SSc. In our study, we applied a composite definition for myocardial involvement based on the opinion of two experts which probably identifies patients with clinically relevant myocardial involvement. The previous study associated elevated TnI with echocardiographic parameters in an SSc population without clear relevant myocardial involvement. Other explanations include a lack of power due to lower than expected values of TnI or inaccuracy of the ELISA kit used for the population under study. However, the ELISA we used had an inter- and intra-assay coefficient of variation <12% and the duplicate measurement confirmed the first measurement in >80% of the samples, so this explanation seems unlikely.

In addition, our study confirmed that elevated TnT levels do not strongly correlate with myocardial involvement. This is in line with a recent review which did not observe an association between cardiac biomarkers (including TnT) and cardiovascular magnetic resonance (CMR) parameters of fibrosis and myocardial involvement and thus concluded that early features of cardiac involvement are not correlated with serologic cardiac biomarkers. Furthermore, this review concluded that biomarkers can point to clinically significant cardiac injury and that, as a consequence, cardiac biomarkers could be useful once cardiac involvement has been diagnosed.^17^ Recently, in a study assessing the characteristics of anti-heart autoantibodies (AHAs) and anti-intercalated disk autoantibodies (AIDAs) (both specific biomarkers of autoimmune myocarditis), both these antibodies correlated with the presence of clinically suspected heart involvement. In this study, clinically suspected heart involvement was defined as having symptoms, an abnormal ECG, abnormal TnI or natriuretic peptides, and abnormal echocardiography. These authors also concluded that there is probably a high burden of autoimmune myocardial involvement in SSc that is underdiagnosed, since they found relatively high frequencies of AHA and AIDA as compared to control groups consisting of non-inflammatory cardiac disease, ischemic heart failure, and healthy donors.^20^ This hypothesis is also in line with previous histopathologic postmortem studies showing myocardial involvement in >70% of SSc patients.^10^ One might hypothesize that the high proportion of SSc samples positive for TnI as compared to healthy controls in our study indicates presence of subclinical myocardial involvement in SSc patients. To address this hypothesis however, an additional, prospective study would be needed ideally including histologic confirmation of myocardial involvement.

It should be emphasized that a clear validated definition regarding the diagnosis of myocardial involvement in SSc is still lacking.^21^ In this study, the definition of myocardial involvement was based on detailed criteria combined with the independent expert opinion of an experienced cardiologist and rheumatologist. One might hypothesize that specific manifestations of myocardial involvement associate differently with troponins. In this study, we are underpowered to perform a sensitivity analysis to evaluate the association between TnI and different manifestations of myocardial involvement. In addition, it would be interesting to evaluate serial TnI measurements where gradual increase of TnI could possibly reflect (development of) myocardial involvement.

This study should be interpreted within its limitations. First, as this study was designed as a cross-sectional study, follow-up data were not included. The subgroup of SSc patients without any clinical organ involvement included patients with increased levels of troponins. It would be interesting to see if these patients will develop myocardial involvement over time, which would demonstrate a potential prognostic value of troponins. Indeed, in a recent paper by Paik et al.,^22^ SSc patients with elevated TnI levels had a 2.16-fold increased risk of death even after correcting for age, sex, disease duration, and cardiopulmonary risk factors. In our study population, we also observed a slightly higher percentage of deaths in patients with elevated TnI group (n = 7 (12.5%) vs n = 2 (8.3%)), but our study is underpowered test to this observation for statistical significance.

For this purpose, we additionally looked into the medical records of the four patients without organ involvement but with TnI levels >20 ng/L. Interestingly, none of these patients have developed clinically relevant myocardial involvement (mean follow-up time 4.5 years).

Second, as commented on before, the sample size of this study might have prevented detection of smaller differences in TnI levels between the different subgroups of SSc patients. Especially in the comparison between patients and healthy donors, the group of healthy donors was relatively small. However, from a diagnostic point of view, smaller differences are of less value as these would complicate diagnostic interpretation in the individual patient.

In conclusion, based on this study, we do not identify an additive value of TnI in the diagnosis of myocardial involvement in SSc patients. Measurement of TnI can still be of prognostic value, but this needs to be evaluated in future studies on prediction of outcome in SSc. As SSc-related myocardial involvement is a poor prognostic factor, and since the prevalence of myocardial involvement in SSc is potentially underestimated, further research focusing on biomarkers for the (early) diagnosis of myocardial involvement is essential.

## Supplemental Material

sj-pdf-1-jso-10.1177_23971983241255550 – Supplemental material for Troponin I levels in systemic sclerosis patients with myocardial involvement Supplemental material, sj-pdf-1-jso-10.1177_23971983241255550 for Troponin I levels in systemic sclerosis patients with myocardial involvement by Eva M Hoekstra, Sophie IE Liem, Saad Ahmed, Nivine Levarht, Cynthia M Fehres, Adrian Giuca, Nina Ajmone Marsan, Tom WJ Huizinga and Jeska K de Vries-Bouwstra in Journal of Scleroderma and Related Disorders
