# Supplementary material for: Troponin I levels in systemic sclerosis patients with myocardial involvement
Source: J Scleroderma Relat Disord. 2024 Jun 3;9(3):185–91. doi: 10.1177/23971983241255550 (PMC11528562; doi:10.1177/23971983241255550)
Supplement: sj-pdf-1-jso-10.1177_23971983241255550 – Supplemental material for Troponin I levels in systemic sclerosis patients with myocardial involvement [file sj-pdf-1-jso-10.1177_23971983241255550.pdf]

Supplementary Table S1. Patient characteristics of included patients with systemic sclerosis at time of sample collection.

	**Myocardial involvement (n=20)**	**Myositis (n=20)**	**No organ involvement (elevated TnT) (n=20)**	**No organ involvement (normal TnT) (n=20)**
Age (years)	54.6 ± 13.8	51.1 ± 11.7	66.2 ± 11.1	62.8 ± 10.2
Female sex, *n (%)*	13 (65.0)	9 (45.0)	12 (60.0)	15 (75.0)
Smoking (at time of study visit), *n (%)*	1 (5.0)	1 (5.0)	4 (20.0)	4 (20.0)
Comorbidity:				
- Cardiovascular disease, *n (%)*	7 (35.0)	2 (10.0)	3 (15.0)	2 (10.0)
- Diabetes mellitus, *n (%)*	1 (5.3)	2 (10.0)	1 (5.0)	1 (5.0)
Disease duration[a] (years)	6.6 (1.3-14.0)	1.5 (0.6-4.6)	4.7 (3.0-12.2)	7.1 (2.8-1.2)
Diffuse SSc, *n (%)*	16 (80.0)	12 (60.0)	4 (20.0)	4 (20.0)
ANA, *n (%)*	18 (90.0)	20 (100)	20 (100)	20 (100)
- Anti-topoisomerase I, *n (%)*	8 (40.0)	5 (25.0)	4 (20.0)	3 (15.0)
- Anti-centromere, *n (%)*	0 (0)	1 (5.0)	6 (30.0)	14 (70.0)
Troponin T (ng/L)[b]	26.0 (10.5-108.3)	122.0 (17.5-457.5)	31.5 (23.5-46.0)	7.0 (4.0-10.0)
Creatinine (µmol/L)	80.0 (58.0-86.0)	69.0 (53.0-77.0)	90.5 (67.8-138.8)	80.0 (71.5-103.3)
CK (U/L)	103.0 (72.0-283.0)	483.0 (157.0-803.0)	123.0 (87.3-146.8)	72.5 (63.3-99.8)
ILD[c], *n (%)*	4 (20.0)	7 (35.0)	1 (5.0)	0 (0)
PAH, *n (%)*	1 (5.0)	1 (5.0)	0 (0)	0 (0)
Use of any immunosuppressives ever[d], *n (%)*	13 (65.0)	19 (95.0)	11 (55.0)	12 (60.0)

Parametric data are reported as mean ± SD, non-parametric data as median (IQR).
[a] Since first non-Raynaud symptom.
[b] Note that 40 out of 80 patients were selected partially based on elevated or normal troponin T levels.
[c] Defined as ILD on CT combined with FVC < 80%.
[d] Includes methotrexate, azathioprine, cyclophosphamide, mycophenolate mofetil, corticosteroids, biologicals.
Abbreviations. ANA: anti-nuclear antibodies; ATA: anti-topoisomerase antibodies; ACA: anti-centromere antibodies; CK: creatinine kinase; ILD: interstitial lung disease; PAH: pulmonal arterial hypertension.

Supplementary Data S1. Score form myocardial involvement.

**Signs of myocardial involvement**	**Yes**	**No**
Conduction abnormality	☐ LBTB<br>☐ RBTB<br>☐ First degree AV block<br>☐ Second degree AV block<br>☐ Third degree AV block<br>☐ Other	☐
Arrhythmias	☐ Atrial fibrillation<br>☐ Ventricular fibrillation<br>☐ > 7% VT<br>☐ > 7% SVT<br>☐ Stops/pause > 3 seconds<br>☐ Other	☐
Pericardial effusion	☐ 0-10mm<br>☐ 10-20mm<br>☐ > 20mm<br>☐ Other	☐
Diastolic dysfunction	☐ Grade 1<br>☐ Grade 2<br>☐ Grade 3<br>☐ End stage<br>☐ Other	☐
Systolic dysfunction	☐ LVEF 40-50%<br>☐ LVEF 30-40%<br>☐ LVEF < 30%<br>☐ Other	☐
Coronary artery disease	☐ PCI<br>☐ CABG<br>☐ Myocardial infarction<br>☐ Other	☐
Valve disease	☐ Stenosis<br>☐ Insufficiency<br>☐ Other	☐
Increased troponin levels	☐ 14-50 ng/L<br>☐ 50-100 ng/L<br>☐ > 100 ng/L	☐
Increased NT-proBNP levels	☐ 250-350 ng/L<br>☐ 350-500 ng/L<br>☐ >500 ng/L	☐
Increased CK levels	☐ 145-200 U/l<br>☐ 200-400 U/l<br>☐ >400 U/l	☐
MRI scan abnormalities	☐ Fibrosis<br>☐ Hypertrophy<br>☐ Ischemia<br>☐ Inflammation<br>☐ Other	☐
**Primary myocardial** involvement due to systemic sclerosis (yes/no)		
